# Supplementary material for: DECODE: a computational pipeline to discover T cell receptor binding rules
Source: Bioinformatics. 2022 Jun 27;38(Suppl 1):i246–54. doi: 10.1093/bioinformatics/btac257 (PMC9235487; doi:10.1093/bioinformatics/btac257)

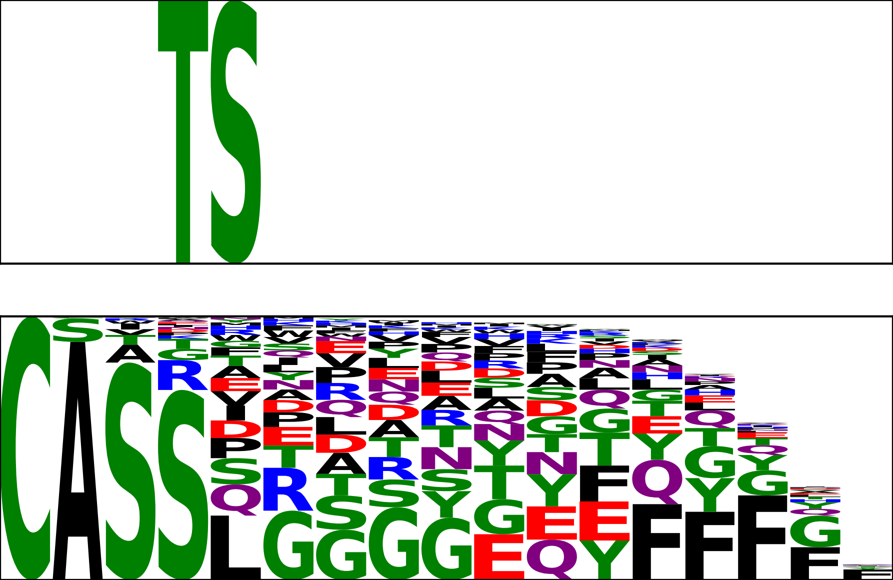

Supplement: btac257_Supplementary_Data [file btac257_supplementary_data.zip › btac257-suppl_data/Nguyen.100.sup.2.zip!/supplementary_data/pMTnet/CDR3_compressed/logo_split1_centroid1061_regionCDR3_compressed3.pdf]
